# Analysis of Right Ventricular Myocardial Stiffness and Relaxation Components in Children and Adolescents With Pulmonary Arterial Hypertension

**DOI:** 10.1161/JAHA.118.008670

**Published:** 2018-04-19

**Authors:** Yasunobu Hayabuchi, Akemi Ono, Yukako Homma, Shoji Kagami

**Affiliations:** ^1^ Department of Pediatrics Tokushima University Tokushima Japan

**Keywords:** diastolic function, diastolic heart failure, pulmonary hypertension, relaxation, right ventricle, right ventricular function, right ventricular pressure overload, stiffness, Pulmonary Hypertension, Clinical Studies, Pathophysiology, Pediatrics, Heart Failure

## Abstract

**Background:**

The rate of left ventricular pressure decrease during isovolumic relaxation is traditionally assessed algebraically via 2 empirical indices: the monoexponential and logistic time constants (τ_E_ and τ_L_). Since the pattern of right ventricular (RV) pressure decrease is quite different from that of the left ventricular, we hypothesized that novel kinematic model parameters are more appropriate and useful to evaluate RV diastolic dysfunction.

**Methods and Results:**

Eight patients with pulmonary arterial hypertension (age 12.5±4.8 years) and 20 normal subjects (control group; age 12.3±4.4 years) were enrolled. The kinematic model was parametrized by stiffness/restoring Ek and damping/relaxation μ. The model predicts isovolumic relaxation pressure as a function of time as the solution of d^2^P/dt^2^+(1/μ)dP/dt+EkP=0, based on the theory that the pressure decay is determined by the interplay of inertial, stiffness/restoring, and damping/relaxation forces. In the assessment of RV diastolic function, τ_E_ and τ_L_ did not show significant differences between the pulmonary arterial hypertension and control groups (46.8±15.5 ms versus 32.5±14.6 ms, and 19.6±5.9 ms versus 14.5±7.2 ms, respectively). The pulmonary arterial hypertension group had a significantly higher Ek than the control group (915.9±84.2 s^−2^ versus 487.0±99.6 s^−2^, *P*<0.0001) and a significantly lower μ than the control group (16.5±4.3 ms versus 41.1±10.4 ms, *P*<0.0001). These results show that the RV has higher stiffness/elastic recoil and lower cross‐bridge relaxation in pulmonary arterial hypertension.

**Conclusions:**

The present findings indicate the feasibility and utility of kinematic model parameters for assessing RV diastolic function.

**Clinical Perspective**

**What Is New?**

- Although empirical parameters such as the monoexponential time constant τ_E_ or the logistic time constant τ_L_ are used to quantitate left ventricular isovolumic pressure decreases, these parameters are not suitable for estimating right ventricular pressure decrease.
- In order to assess right ventricular diastolic dysfunction in patients with pulmonary arterial hypertension, we investigated the feasibility and usefulness of the kinematic model parameters based on the theory that the pressure decay is determined by the interplay of inertia, stiffness/restoring, and damping/relaxation.

**What Are the Clinical Implications?**

- The pulmonary arterial hypertension group has higher stiffness/restoring Ek and lower cross‐bridge relaxation μ than the control group.
- This study indicates the validation of kinematic model parameters for assessing right ventricular diastolic function in patients with pulmonary arterial hypertension.

Patients with pulmonary arterial hypertension (PAH) ultimately develop right heart failure.^1^ Previous studies have demonstrated that patients with PAH have reduced systolic function as measured by right ventricular (RV) ejection fraction. Although most clinical research has focused on systolic function, normal RV filling is also essential to maintain exercise activity and adapt to acute and chronic overload. However, knowledge of the role of RV diastolic function in PAH is limited.^2^, ^3^ Abnormalities in both active cross‐bridge relaxation and passive elastic recoil are observed in the hypertrophied RV myocardium, eventually leading to RV diastolic dysfunction, which results in increases in RV filling and right atrial pressures. Indeed, they are associated with disease progression and increased mortality in both adults and children with PAH.^4^, ^5^, ^6^ However, assessment of RV diastolic function is challenging.^2^, ^3^ Consequently, few studies have investigated RV diastolic function, particularly in pediatric patients with PAH.^7^ Accurate measurement of RV diastolic function could contribute to improved clinical management of these patients.

The time constant (τ) is considered the best empirical standard for estimating the rate of pressure decrease in the assessment of left ventricular (LV) diastolic function.^8^, ^9^ However, the pattern of RV pressure decrease is quite different from that of LV pressure decrease.^10^ The peak rate of pressure decrease (dP/dt_min) is not a reliable reference point for evaluating the onset of RV diastole, because it appears when the major portion of RV pressure decrease has already occurred.^10^ The time constant (τ) evaluates a relatively much shorter segment in the RV than in the LV. The LV pressure decay model proposed by Chung and Kovács uses Newton's second law in accordance with the known chamber shape change during isovolumic relaxation (IVR).^11^, ^12^ It is a kinematic model that predicts IVR pressure from before dP/dt_min to near mitral valve opening. Pressure decay is determined by the cross‐bridge uncoupling, elastic recoil/restoring forces, and inertial forces. The model is parametrized by stiffness/restoring Ek and damping/relaxation μ.^11^

We hypothesized that these physical and physiological principles govern IVR pressure of the RV, and the mathematical model correctly quantifies the pathological RV diastolic dysfunction in PAH in children and adolescents.

## Methods

The data, analytic methods, and study materials will not be made available to other researchers for purposes of reproducing the results or replicating the procedure.

### Study Population

The participants in this prospective study were 8 consecutive pediatric patients (mean age±SD, 12.5±4.8 years; age range, 6–20 years) with PAH (PAH group). All patients had been scheduled for evaluations of their circulatory condition. The patients’ conditions were as follows: idiopathic PAH (n=6); idiopathic PAH with a coincidental small atrial septal defect (n=1) and a small ventricular septal defect (n=1). Furthermore, 20 consecutive subjects (mean age, 12.3±4.4 years; age range, 6–20 years) whose LV and RV pressures, volumes, and function were assessed as normal were enrolled in this study (control group). The control group consisted of patients with the following diagnoses: 9 patients after Kawasaki disease without any coronary arterial stenosis or myocardial ischemia; 9 patients with patent ductus arteriosus with Qp/Qs <1.1, for whom catheter occlusion was planned; and 2 patients who had concealed Wolf‐Parkinson‐White syndrome and who underwent catheter ablation. Data collected from December 2013 to December 2016 were analyzed. All study protocols conformed to the ethical guidelines of the Declaration of Helsinki (1975) and were approved by the Institutional Review Board of Tokushima University Hospital. Written, informed consent for their children to participate in the study was provided by the parents.

### Cardiac Catheterization

Cardiac catheterization and angiography (Integris Allura 9 Biplane; Phillips Medical Systems, Best, The Netherlands) proceeded using 4‐ to 6‐Fr catheters. Data were acquired during routine cardiac catheterization. LV and RV pressure measurements were performed using a high‐fidelity manometer‐tipped 0.014‐inch pressure wire (PressureWire Aeris; St. Jude Medical, Inc, St. Paul, MN). Recordings were made with respiration suspended at the end of expiration. All hemodynamic data were acquired at a sampling rate of 100 Hz before the administration of any contrast agents.

**Figure 1:**
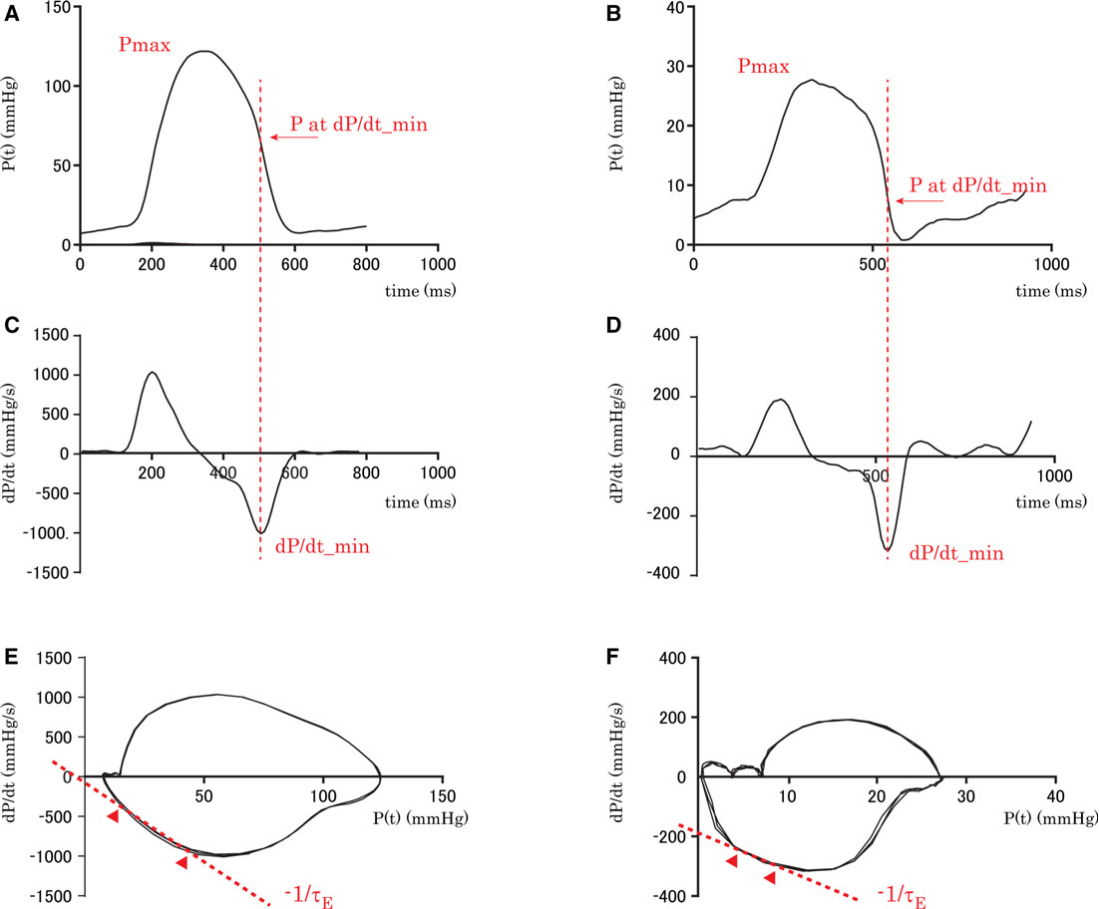
Representative example of time courses of left ventricular (LV) pressure (A), right ventricular (RV) pressure (B), LV dP/dt (C), and RV dP/dt (D). The vertical red dotted line marks the times at dP/dt_min of the LV and RV. LV dP/dt_min occurs when LV pressure falls to 54% of its peak‐systolic pressure (Pmax). RV dP/dt_min occurs when RV pressure falls to 33% of Pmax. The pressure phase planes (PPPs; dP/dt vs P) of LV (E) and RV (F) are shown. The negative inverse of the slope of the isovolumic pressure decrease shown by the red dotted lines in the PPP indicates monoexponential τ. The time interval for the calculation of the time constant is shown between the triangles. Note that the time interval of RV is much shorter than that of LV. dP/dt indicates time derivatives of pressure; P(t), time‐varying pressure.

For each subject, the time‐varying pressure (P(t)) (Figure 1A and 1B), the time derivatives of pressure (dP/dt) (Figure 1C and 1D), LV and RV end‐diastolic pressures (LVEDP and RVEDP), maximum and minimum pressures and pressure derivatives (dP/dt_max, and dP/dt_min), and the IVR pressure inflection point were determined. The pressure phase plane (PPP), where dP/dt is plotted against P(t), was delineated (Figure 1E and 1F).^13^, ^14^, ^15^ LVEDP was defined by the LV pressure at the ECG R‐wave peak. The mitral valve opening and tricuspid valve opening times were determined as the time points where the decaying pressure contours were closest to the LVEDP and RVEDP, respectively, of the subsequent filling beat.^16^, ^17^

### Assessment of Monoexponential and Logistic Time Constants

Diastolic function has traditionally been evaluated using the IVR time constant, which describes the pressure decrease. In the monoexponential model of pressure decay, it is assumed that the time derivative of pressure decay is proportional to pressure. The governing differential equation for the monoexponential model is

(1A) $$\tau_{E}\frac{\text{dP}(t)}{\text{dt}}+\left(P_{0}-P_{\infty }\right)e^{-t/\tau_{E}}=0$$

or

(1B) $$P\left(t\right)=\left(P_{0}-P_{\infty }\right)e^{-t/\tau_{E}}+P_{\infty }$$

where τ_E_ is the monoexponential time constant of IVR pressure, and P_∞_ is the pressure asymptote.^8^, ^9^ A convenient way to determine it is to plot Eq. (1A)A in the PPP, where a straight line with a slope of −1/τ_E_ and an intercept on the dP/dt axis is inscribed, and it is then fit to the IVR portion of the loop that is inscribed by P(t) for the cardiac cycle (Figure 1E and 1F).^14^, ^15^ However, because there are curvilinear IVR segments, a straight‐line fit to the IVR portion of the loop is not always physiological (Figure 1F). In addition, RV pressure decay in particular has been shown to have curvilinear IVR segments.^10^ Thus, τ_E_ may not be suitable for evaluating RV diastolic function.

Another empirical constant has been proposed as an alternative to fit these common curved IVR segments of PPP trajectories.^15^ The logistic time constant τ_L_, which is similar to τ_E_, provides an empirical fit, in which the rate of pressure decrease is proportional to the square of the pressure and is given by

(2A) $$\frac{P^{2}}{P_{A}}+\tau_{L}\frac{\text{dP}}{\text{dt}}+P\left(t\right)-P_{B}=0$$

or

(2B) $$P\left(t\right)=\frac{P_{A}}{1+e^{-t/\tau_{L}}}+P_{B}$$

where τ_L_ is the logistic time‐constant of IVR, and the pressure asymptote is given by the sum of P_A_ and P_B_. This logistic relationship is quadratic in P(t), and it can only produce, and, therefore, best fit, curvilinear PPP IVR contours in the PPP.

The PPP was determined for each beat in each subject. The slope of the dP/dt versus P(t) plot over the interval between 10 ms after dP/dt_min and 10 ms before the estimated mitral valve opening time determined by the least‐squares method was equal to −1/τ_E_.^8^, ^9^, ^13^ τ_L_ was obtained using the methods of Matsubara et al^15^ with a customized Levenberg‐Marquardt algorithm.^18^ Both τ_E_ and τ_L_ were determined using an automated Java program.

### Kinematic Modeling of Ventricular Pressure Decay

Chung and Kovács previously showed that LV pressure decay is accurately determined mathematically by the interactions of inertial, stiffness, and relaxation forces using physiological‐kinematic arguments, and they published their experimental results.^11^ The relative contributions of stiffness and relaxation to IVR pressure are characterized by a stiffness parameter and a damping or relaxation parameter.^19^ In the kinematic model, IVR pressure is predicted from before dP/dt_min to near mitral valve opening. Their theory applies the kinematics of the damping oscillator governed by the (mass normalized) equation of motion:

(3A) $$\frac{d^{2}x}{{\text{dt}}^{2}}+c\frac{\text{dx}}{\text{dt}}+\mathrm{kx}=0$$

where k is stiffness and c is damping.^19^ The parameters of this model are stiffness/restoring Ek and damping/relaxation μ. The equation for LV pressure during this IVR phase is

(3B) $$\frac{d^{2}P}{{\text{dt}}^{2}}+\frac{1}{\mu }\frac{\text{dP}}{\text{dt}}+\mathrm{Ek}\left(P-P_{\infty }\right)=0$$

The solution for this equation in the underdamped regime (1/μ^2^<4Ek) for pressure or for the time derivative of pressure is given by

(4) $$P\left(t\right)=e^{-t/2\mu }\left[\frac{{\dot{P}}_{0}+P_{0}/2\mu }{\omega }\sin\left(\omega \cdot t\right)+P_{0}\cos\left(\omega \cdot t\right)\right]+P_{\infty }$$

(5) $$\frac{\text{dP}}{\text{dt}}\left(t\right)=e^{-t/2\mu }\left[-\left(\frac{{\dot{P}}_{0}/\mu +2E_{k}P_{0}}{2\omega }\right)\sin\left(\omega \cdot t\right)+{\dot{P}}_{0}\cos\left(\omega \cdot t\right)\right]$$

where P_o_ is the initial pressure assuming a zero‐pressure asymptote, ${\dot{P}}_{o}$ is the initial time derivative of pressure, and

$$\omega =\sqrt{4\text{Ek}-1/\mu^{2}}/2$$

The critically damped (1/μ^2^=4Ek) and overdamped (1/μ^2^>4Ek) solutions are provided by evaluating Eqs. 4 and 5 at the ω=0 (critically damped) or ω=iβ (overdamped) limits.

When 1/μ^2^=4Ek (critically damped kinematics):

(6) $$P\left(t\right)=P_{0}e^{-t/2\mu }+\left({\dot{P}}_{0}+P_{0}/2\mu \right)e^{-t/2\mu }t+P_{\infty }$$

When 1/μ^2^>4Ek (overdamped kinematics):

(7) $$P\left(t\right)=e^{-t/2\mu }\left[\frac{{\dot{P}}_{0}+P_{0}/2\mu }{\beta }\sinh\left(\beta \cdot t\right)+P_{0}\cosh\left(\beta \cdot t\right)\right]+P_{\infty }$$

$$\beta =\sqrt{1/\mu^{2}-4\text{Ek}}/2$$

To extract Ek and μ from an isovolumic pressure contour, which is the equivalent to solving the “inverse problem of IVR pressure,” the procedure is as follows.

**Figure 2:**
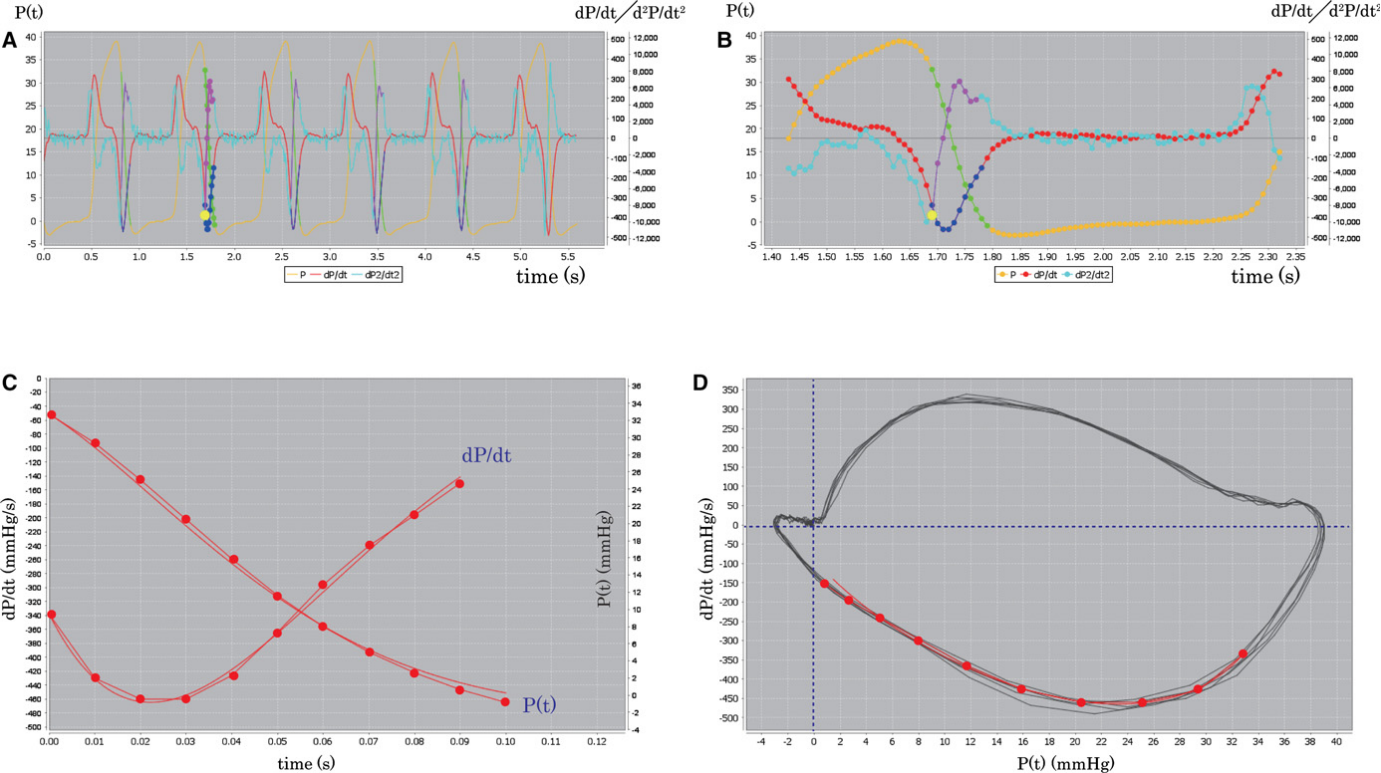
Appearance of the custom‐automated Java program (Pressure Decay Analysis Tokushima [PDA‐Tokushima] ver. 1.05) for the calculation of the kinematic model parameters. Pressure, dP/dt, and d^2^P/dt^2^ of several cardiac cycles are shown (A). One cardiac cycle of these waves is shown as an enlarged display (B). The actual measured pressure and dP/dt are shown as dots, and fitting curves for P and dP/dt obtained from equations (4) and (5) are shown as the curvilinear lines (C). PPP trajectories are shown by the black curvilinear portion. The actual measurements are shown by the red dots. The calculated kinematic model fitting curve using Ek and μ is shown by the red curvilinear portion (D). dP/dt indicates time derivatives of pressure; PPP, pressure phase planes; P(t), time‐varying pressure.

With a custom‐automated Java program (Pressure Decay Analysis Tokushima [PDA‐Tokushima] ver. 1.05), hemodynamic data were analyzed (Figure 2). Ek, μ, P_o_, and ${\dot{P}}_{o}$ were extracted for each IVR pressure contour in each subject via Eq. (5) from dP/dt versus t data by a Levenberg‐Marquardt fitting algorithm to the dP/dt data.^11^, ^20^ The initial point for the fitting was from the inflection point in the IVR pressure contour before dP/dt_min, while the end point was taken to be 10 ms before the estimated mitral valve opening or tricuspid valve opening time. Having found Ek, μ, P_o_, and ${\dot{P}}_{o}$, Eq. (4) was used to determine P with the Levenberg‐Marquardt algorithm and the other 4 parameters held constant. Since IVR pressure contours are nonphysiological and noisy, they generate high root mean square error values between the raw dP/dt data and the model fit dP/dt when they are compared with acceptable physiological data. Therefore, beats having the largest 50th percentile root mean square error values were discarded. Thus, only physiological smooth data were included in the final analysis, and this had the additional advantage of being automated, which minimized observer bias in beat selection. Finally, each subject's parameters were determined by selecting and averaging 5 beats.

### Influence of Preload on the Diastolic Functional Parameters

The influence of preload on the diastolic functional parameters was also assessed. Data measured during an increase in venous return by abdominal compression were used to determine the influence of preload.^21^, ^22^ RV pressure was measured this way in 15 subjects.

### Statistical Analysis

All data are expressed as means±SD or as medians with the 5th to 95th percentiles. The significance of differences was determined using the Mann–Whitney *U* test or the Kruskal–Wallis test followed by Dunn's test, as appropriate. Linear regression analyses were performed for the correlations, and Pearson's correlation coefficients were calculated. All statistical data were analyzed using Prism (version 6.0; GraphPad Software, San Diego, CA) and JMP 11 (SAS Institute, Inc, Cary, NC). *P*<0.05 (2‐sided) were considered significant. Intraobserver variability was assessed by 1 investigator (Y.H.) conducting measurements on the same patients 8 weeks apart, and interobserver variability was assessed by a second investigator (A.O.) who was unaware of the previous results and performed the same measurements on 10 randomly selected participants. Intraobserver and interobserver agreements were assessed using intraclass correlation coefficients (ICCs). In addition, agreement between investigators was tested using Bland‐Altman analysis by calculating the bias (mean difference) and 1.96 SD around the mean difference.

## Results

No subjects were excluded from the analysis because of suboptimal pressure recordings. Therefore, the study population comprised 8 subjects with PAH (PAH group; mean age±SD, 12.5±4.8 years; age range, 5–20 years) and 20 subjects with normal LV and RV functions (control group; 12.3±4.4 years; 5–20 years). The participants’ clinical and hemodynamic data, along with ranges, are shown in Table 1.

**Table 1:** Subjects’ Clinical Characteristics

	Control (n=20)	PAH (n=8)	*P* Value
Sex (male/female)	9/11	3/5	0.7171
Age, y	12.3±4.4 (5–20)	12.5±4.8 (5–20)	0.9164
Weight, kg	39.6±14.2 (17.1–67.0)	40.5±15.3 (20.2–61.1)	0.8832
Height, cm	142.4±21.8 (110.0–172.2)	145.9±19.7 (114.0–171.0)	0.6971
Body surface area, m^2^	1.24±0.32 (0.70–1.79)	1.28±0.33 (0.78–1.67)	0.7694
Heart rate, bpm	86±17 (59–120)	84±16 (66–105)	0.7774
Systolic blood pressure, mm Hg	100±15 (75–128)	100±14 (80–120)	0.9999
Diastolic blood pressure, mm Hg	56±10 (38–70)	56±9 (39–67)	0.9999
RVSP, mm Hg	19±3 (14–25)	55±14 (43–85)	<0.0001
RVEDP, mm Hg	4±2 (1–8)	10±2 (7–14)	<0.0001
MPAP, mm Hg	11±3 (7–16)	40±13 (27–62)	<0.0001
RVEF, %	63±5 (55–79)	41±5 (35–47)	<0.0001
Time on treatment, y		5.1±3.7 (1–12)	
Treatment		Epoprostenol 2 Bosentan 3 Macitentan 5 Tadalafil 8	

Data are shown as means±SD and range in parentheses. bpm indicates beats per minute; MPAP, mean pulmonary arterial pressure; PAH, pulmonary arterial hypertension; RVEDP, right ventricular end‐diastolic pressure; RVEF, right ventricular ejection fraction; RVSP, right ventricular systolic pressure.

### RV and LV Pressure Decreases in Normal Subjects

Representative examples of cardiac cycles in the control group are shown as LV and RV pressures, dP/dt time courses, and PPP (Figure 1). The pattern and rate of RV pressure decay can be compared with those of LV pressure decay. Both ventricles show 2 distinct phases of pressure decrease: an initial accelerative phase and a subsequent decelerative phase separated by the corresponding dP/dt_min. In the LV, the initial accelerative phase (until LV‐dP/dt_min) encompassed 25.05±3.1%, while the major part of the LV pressure decreased during the subsequent phase in a decelerative fashion. In contrast, the accelerative phase of the RV was 67.5±4.9% of its course, significantly shorter than that of the LV (*P*<0.001). The dP/dt_min of the LV and RV were −1013.7±188.1 mm Hg/s and −402.2±165.3 mm Hg/s, respectively (*P*<0.0001). The ratio of pressure at dP/dt_min to maximum pressure (P at dP/dt_min/maximum pressure) was significantly lower in the RV than in the LV (28.6±14.8% and 58.3±7.5%, respectively; *P*<0.0001).

### Kinematic Model Validation

Model validation was assessed in all 28 participants (Table 2). Statistically, the kinematic model‐predicted contour provided the best and most consistent fit to the IVR portion in LV pressure decay. Furthermore, the kinematic model fit also consistently had lower root mean square errors in both P versus t and dP/dt versus t in the assessment of the RV, indicating that it can be a more physiologically accurate model of IVR pressure.

**Table 2:** RMSEs of the Evaluated Parameters Versus Actual Data

Parameter	LV	RV	*P* Value
Monoexponential τ (τ_Ε_)			
RMSE of P vs t, mm Hg	1.47±0.56	1.78±0.75	0.0354
RMSE of dP/dt vs t, mm Hg/s	70.2±19.2	85.3±19.4	0.0050
Logistic τ (τ_L_)			
RMSE of P vs t, mm Hg	1.39±0.59	1.18±0.45^a^	0.0401
RMSE of dP/dt vs t, mm Hg/s	75.3±21.9	55.1±17.5^b^	0.0114
Kinematic model (Ek and μ)			
RMSE of P vs t, mm Hg	0.88±0.25^c^	0.90±0.31^c^	0.7915
RMSE of dP/dt vs t, mm Hg/s	41.2±14.2^c^	50.3±15.5^d^	0.0259

Data are means±SD of the RMSEs of the evaluated parameters vs actual data in the P vs t or dP/dt vs t plane. dP/dt indicates time derivatives of pressure; LV, left ventricular; RMSE, root mean squared error; RV, right ventricular.

a *P*=0.0106 vs monoexponential τ.

b *P*=0.0011 vs monoexponential τ.

c *P*<0.0001 vs monoexponential τ, and *P*<0.0001 vs logistic τ.

d *P*<0.0001 vs monoexponential τ, and *P*=0.0282 vs logistic τ.

### Diastolic Parameters of the LV and RV in the Control Group

**Figure 3:**
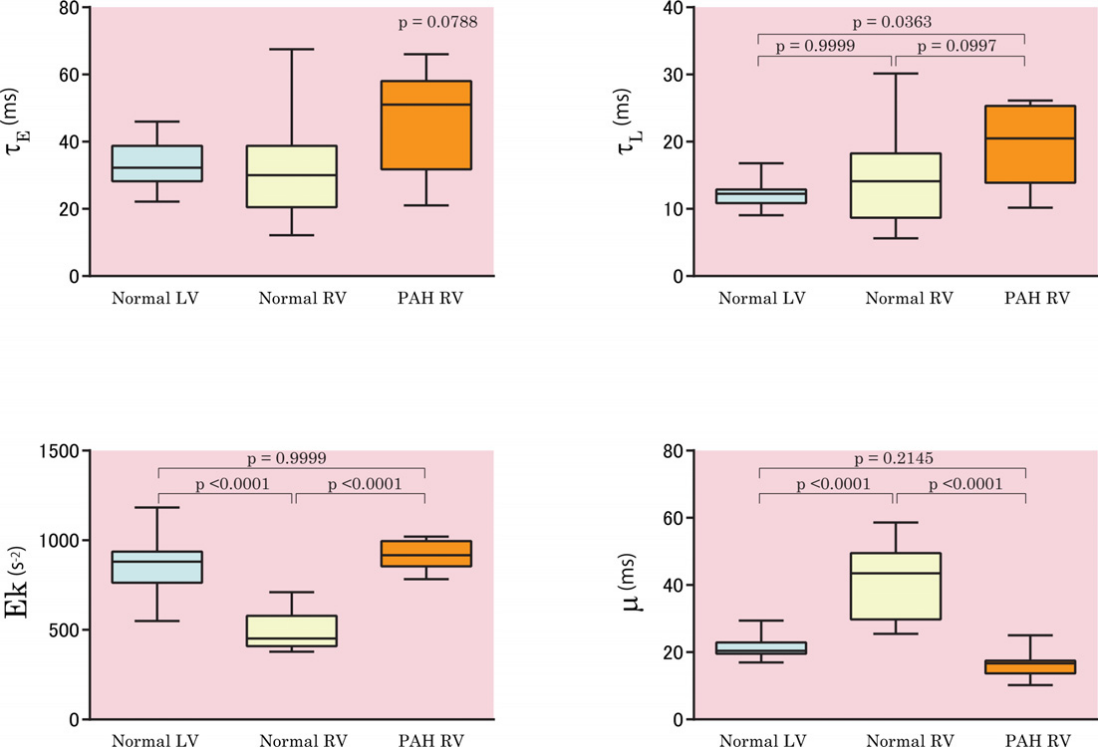
The values of τ_E_, τ_L_, Ek, and μ of the normal LV, normal RV, and RV in the PAH group are shown. Boxes, IQR; central line, median. Whiskers, 5th to 95th percentiles. LV indicates left ventricular; PAH, pulmonary arterial hypertension; RV, right ventricular; τ_E_, monoexponential time constant; τ_L_, logistic time constant.

The obtained parameter values for τ_E_, τ_L_, and the kinematic model parameters Ek and μ were compared between the LV and RV in the control group to assess the characteristics of normal RV diastolic physiology. Thereafter, the results obtained from RV pressure in the PAH group were compared with those of the normal RV to elucidate the RV diastolic pathophysiology in pressure overload (Figure 3).

In the control group, τ_E_ and τ_L_ were not significantly different between the LV and RV (33.1±6.9 ms versus 32.5±14.6 ms, and 12.6±2.4 ms versus 14.5±7.2 ms, respectively). Furthermore, τ_E_ and τ_L_ of the PAH RV were 46.8±15.5 and 19.6±6.0 ms, respectively, and they were not significantly different from those of the normal RV. In the analysis of the kinematic model, Ek was significantly lower in the normal RV than in the normal LV (487.0±99.6 s^−2^ versus 858.1±162.7 s^−2^, *P*<0.0001), whereas μ was significantly higher in the RV than in the LV (41.1±10.4 ms versus 21.5±3.8 ms, *P*<0.0001). These results indicate that the normal RV has lower stiffness/elastic recoil and superior cross‐bridge relaxation.

### Diastolic Parameters of the RV in the PAH Group

Measured τ_E_ and τ_L_ were not significantly different between the PAH and control groups (46.8±15.5 ms versus 32.5±14.6 ms, and 19.6±5.9 ms versus 14.5±7.2 ms, respectively). The PAH group had significantly higher Ek than the control group (915.9±84.2 s^−2^ versus 487.0±99.6 s^−2^, *P*<0.0001) and significantly lower μ than the control group (16.5±4.3 ms versus 41.1±10.4 ms, *P*<0.0001). These results demonstrate that the PAH RV has higher stiffness/elastic recoil and lower active relaxation in diastole.

### Influence of Preload on the Parameters

**Figure 4:**
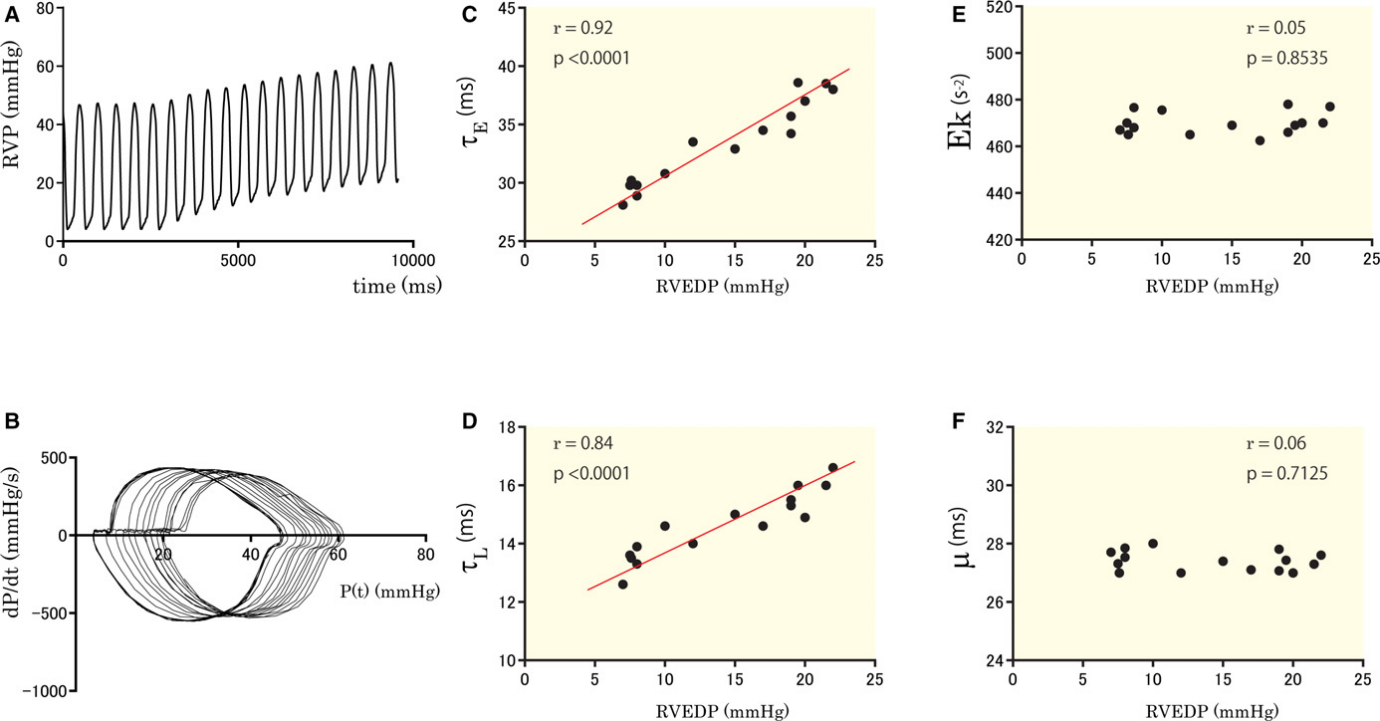
Effect of increased preload on the diastolic functional parameters. Representative recording of RVP with increased preload during abdominal compression (A). PPP loops are shown (B). The changes of τ_E_ (C), τ_L_ (D), Ek (E), and μ (F) vs RVEDP are shown for the data of (A). PPP indicates pressure phase plane; RVEDP, right ventricular end‐diastolic diameter; RVP, right ventricular pressure; τ_E_, monoexponential time constant; τ_L_, logistic time constant.

Next, the influence of increased preload on the RV diastolic functional parameters was examined in 15 subjects (12 control and 3 PAH). Representative RV pressure recordings are shown in Figure 4A and 4B. Significant changes were observed in systolic and end‐diastolic pressures in all subjects who underwent abdominal compression. During the maneuver, RV systolic and end‐diastolic pressures were significantly elevated (30.8±11.5 mm Hg versus 39.2±13.2 mm Hg, *P*<0.001; and 5.0±2.9 mm Hg versus 16.3±4.1 mm Hg, *P*<0.0001, respectively). The changes of diastolic parameters along with the RVEDP in a representative case are shown (Figure 4C through 4F). Significant correlations between parameters and RVEDP were shown in 12, 9, 3, and 2 of 15 cases for τ_E_, τ_L_, Ek, and μ, respectively. These results indicate that the kinematic model parameters are minimally affected by preload alteration.

### Reproducibility

To assess the reproducibilities of the time constants (τ_E_ and τ_L_) and the kinematic parameters (Ek and μ), intra‐ and interobserver variabilities in the measurements were confirmed in 10 randomly selected participants (7 control and 3 PAH) by means of ICCs and Bland‐Altman analysis. The ICCs of Ek were 0.97 and 0.96 for intra‐ and inter‐observer variabilities, respectively. The ICCs of μ for intra‐ and interobserver reproducibilities were 0.96 and 0.95, respectively. On the other hand, the ICCs of τ_E_ for intra‐ and interobserver reproducibilities were 0.81 and 0.85, respectively. For τ_L,_ the ICCs for intra‐ and interobserver reproducibilities were 0.86 and 0.85, respectively.

**Figure 5:**
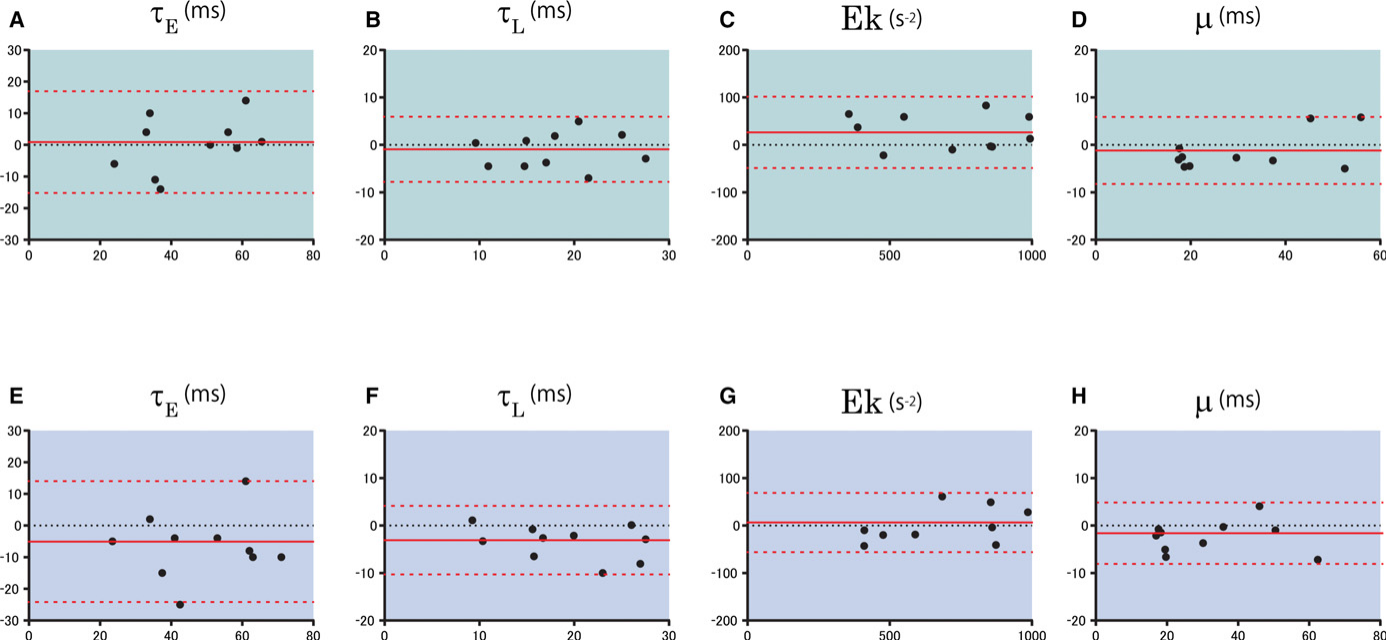
Bland‐Altman plots for the parameters. Intraobserver (A through D) and interobserver (E through H) variabilities are shown. The solid and dotted lines show the bias±1.96 SD (95% limit of agreement). τ_E_, monoexponential time constant; τ_L_, logistic time constant.

Bland‐Altman analysis also showed minimal bias and substantial agreement for reproducibility (Figure 5). Ek and μ measurements proved to be highly reproducible.

## Discussion

The present study demonstrated that the causality‐based kinematic parameters, Ek and μ, could precisely characterize load‐independent RV diastolic function, whereas τ_E_ and τ_L_ of the RV did not show significant differences between the control and PAH groups.

The present results showed that the RV has significantly different diastolic properties, including stiffness/elastic recoil and active relaxation, from the LV. The kinematic model demonstrated that the normal RV has lower passive stiffness/restoring and higher active relaxation than the normal LV. Moreover, the RV in the PAH group was significantly stiffer and had slower cross‐bridge detachment relaxation than the RV in the control group. To the best of our knowledge, this is the first application of kinematic model parameters for the assessment of RV diastolic function. This method was found to be a quite useful way for evaluating RV diastolic dysfunction in patients with PAH. Although some previous reports suggested the usefulness of τ_E_ and τ_L_ for the assessment of RV diastolic dysfunction in patients with PAH,^3^, ^7^, ^23^ this issue has remained controversial. The present results did not show significant differences in these indices between the normal and PAH groups. This discrepancy might result from the subjects’ age, disease duration, and severity. The progression of deterioration of RV diastolic function, which consists of active relaxation and stiffness/elastic recoil, might differ between children and adults.

Furthermore, the RV and LV pressure decreases were found to follow distinct time courses. The initial accelerative phase until dP/dt_min is relatively longer, and the subsequent decelerative phase is shorter in the RV than in the LV. In this respect, the RV time constants τ_E_ and τ_L_ evaluate a quite short segment of RV pressure decay. However, on the molecular level, both τ_E_ and τ_L_ have been shown to correlate with active relaxation as defined by deactivation events, such as cross‐bridge cycling, calcium handing, or lusitropism,^24^, ^25^ but neither can fully characterize the full range of the IVR pressure decrease. We considered that these methods can cause measurement errors in the fitting equation because of the small number of sample points. We should, therefore, be aware that the RV time constant only evaluates a minor portion of the RV pressure decrease and has low reproducibility. When compared with τ_E_ and τ_L_ models, the kinematic model parameters Ek and μ provided a superior fit to IVR pressure and higher reproducibility.

Previous work by Chung and Kovács demonstrated that IVR pressure is precisely determined by the interplay of stiffness/elastic recoil and damping/relaxation forces.^11^ The relative contributions of stiffness and relaxation to IVR pressure decay are characterized by the stiffness/restoring parameter Ek and the damping/relaxation parameter μ. This kinematic model successfully unifies the previously unrelated τ_E_ and τ_L_ models of isovolumic pressure decay in a parametric limit sense. The model proposed in Eq. (3B)B explains why PPP contours can change shape. A linear IVR PPP segment is one where the relaxation parameter (1/μ) is large compared with the elastic term (Ek).^26^ As the elastic term increases, the IVR PPP segment becomes more curvilinear.^26^ A recent study involving humans demonstrated RV hypertrophy with collagen deposition, increased sarcomeric stiffness, and changed titin isoform and phosphorylation.^2^, ^27^ RV diastolic behavior should be evaluated from the perspectives of stiffness and relaxation. In this respect, the kinematic model established the parameters conforming to the pathophysiological state. Furthermore, to more fully characterize the novel parameters, the influence of preload alteration on the parameters was assessed using abdominal compression. The IVR pressure contour has been found to be sensitive to both intrinsic relaxation properties and extrinsic load.^28^, ^29^, ^30^ Indeed, the load dependence of τ_E_ and τ_L_ is well established,^28^, ^29^, ^30^ and, therefore, the variations in τ_E_ and τ_L_ between subjects may be the result of intrinsic chamber property differences or may be caused by extrinsic load effects in the assessment of LV diastolic function. The present data also showed that RV τ_E_ and τ_L_ are significantly correlated with RVEDP. Thus, a load‐independent index that overcomes the limitations of τ_E_ and τ_L_ would be advantageous. The kinematic parameters Ek and μ were relatively independent of preload in the present investigation. Furthermore, Shmuylovich and Kovács applied this kinematic model and derived a load‐independent parameter, named *M*_LIIIVPD_, which is the constant slope between the effective peak elastic recoil forces that drive pressure decline during isovolumic relaxation and the peak resistive forces that oppose cross‐bridge uncoupling and pressure decline.^31^

With the combined pressure conductance catheter, it has become possible to determine ventricular pressure and volume simultaneously. The criterion standard for measuring load‐independent diastolic stiffness by pressure‐volume analysis is not without risk in patients with PAH because it requires temporal preload reduction.^32^, ^33^ In left heart failure, this was circumvented by the development of single‐beat analyses of the diastolic pressure‐volume relationship.^34^, ^35^ However, it is unclear whether this analysis could also be used for the RV in PAH. Furthermore, since precise RV volumetric measurement is challenging, it would be quite difficult to assess RV diastolic function using pressure‐volume analysis. Doppler echocardiography is the preferred method for noninvasive diastolic function assessment. Previous studies modeled filling in kinematic terms via the parameterized diastolic filling formalism.^36^, ^37^ This model characterizes transmitral blood flow velocity in terms of elastic, inertial, and damping forces. During filling, the elastic driving force generates both inertial forces, causing acceleration, and resistive (damping) forces, opposing acceleration. The 3 mathematically independent model parameters—spring constant, damping constant, and initial spring displacement—fully characterize the velocity of the E‐wave.^36^, ^37^ The transmitral flow‐based load‐independent index of diastolic function can be derived and validated for the LV.^38^

In the present study, it was, therefore, demonstrated that the kinematic model parameters have high reproducibility and can be determined independent of volume. Diastolic dysfunction determines ventricular performance and patient outcomes in many conditions, and it may precede systolic dysfunction.^23^ We concluded that this method has great clinical implications for the management of patients with PAH.

### Study Limitations

It is necessary to be aware of the technical problem that the small number of sample points for pressure measurement results in inaccurate parameter estimation. In the comparison of root mean square error between τ_E_, τ_L_, and the kinematic model, our approach may be criticized because 2 model parameters, μ and Ek, can always provide a better curve fit to data than a single parameter such as τ_E_ and τ_L_. Although a model with 2 free parameters is in general always better than a model with 1 free parameter when performing conventional curve fitting to data points, the necessity for 2 parameters was dictated by modeling the physics and physiology in elastic recoil and relaxation terms.

The aim of the present study was to establish the kinematic parameters as RV diastolic functional indices, and they were validated by evaluating normal LV, normal RV, and PAH RV diastolic functions. Thus, the study design did not analyze the relationships between the parameter values and PAH severity, including mean pulmonary arterial pressure, right ventricular systolic pressure, and RVEDP. Since the present study population was small, and the patients’ clinical courses and treatment were heterogeneous, such detailed analysis would not be meaningful. Further studies are needed to determine whether these parameters could serve as useful evaluation tools and become the criterion standard for assessing RV diastolic function and to predict the prognosis of patients with this disease.

## Conclusions

The present findings suggest the feasibility and usefulness of kinematic model parameters for evaluating RV diastolic function. This method is based on the pathophysiological theory, is load‐independent, and is highly reproducible.

## Disclosures

None.
